# Hard-X-ray Zone Plates: Recent Progress

**DOI:** 10.3390/ma5101752

**Published:** 2012-09-27

**Authors:** Syue-Ren Wu, Yeukuang Hwu, Giorgio Margaritondo

**Affiliations:** 1 Department of Engineering and System Science, National Tsing Hua University, Hsinchu 300, Taiwan; E-Mail: hjwu@phys.sinica.edu.tw; 2 Institute of Physics, Academia Sinica, Taipei 115, Taiwan; 3 School of Basic Sciences, Ecole Polytechnique Fédérale de Lausanne (EPFL), Lausanne CH-1015, Switzerland; E-Mail: giorgio.margaritondo@epfl.ch

**Keywords:** X-ray microscopy, radiology, Fresnel zone plates, X-ray focusing, synchrotron radiation, free electron laser, microtomography

## Abstract

The technology to focus hard-X-rays (photon energy larger than 1–2 keV) has made great progress in the past three years. The progress was particularly spectacular for lenses based on the Fresnel zone plate concept. The spatial resolution notably increased by a factor of three, opening up entirely new domains of application, specifically in biomedical research. As we shall see, this evolution is the result of a painstaking optimization of many different aspects rather than of a single technical breakthrough.

## 1. Background

The technical evolution of zone plate devices for hard X-rays dramatically accelerated in the past two years [1,2,3,4,5,6,7,8,9,10,11,12,13,14,15,16,17,18,19,20,21,22,23,24,25,26,27,28,29]. Milestones like a lateral resolution below 10 nm, formerly a dream, appear now realistic [1]. This is primarily a response to the needs created by the equally spectacular progress in X-ray sources [30,31]. As a consequence, X-ray imaging and spectromicroscopy increasingly impact new research domains, mostly—but not only—in the life sciences [1].

Developing optical devices for the X-ray spectral range is a challenging technological task. In the visible range, such devices exploit phenomena like reflection and refraction. These phenomena are quite different between X-rays and visible light.

**Figure 1:**
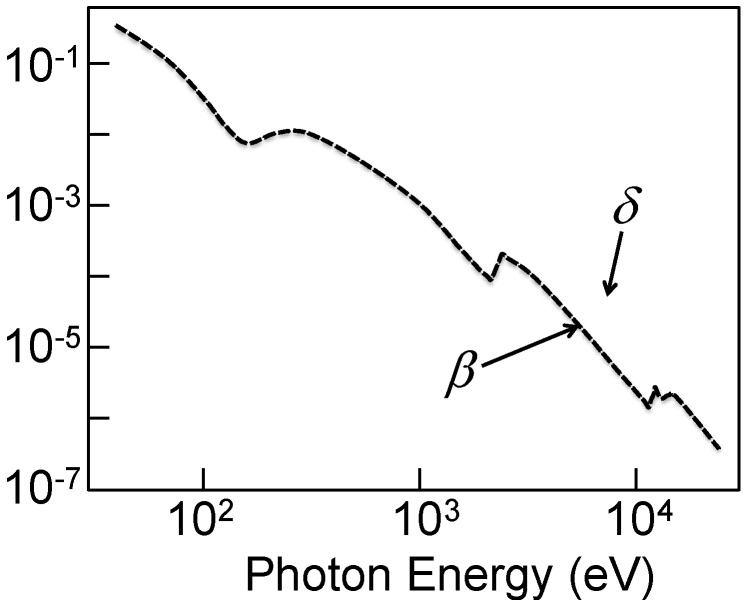
The *δ* and *β* parameters of the index of refraction of gold, plotted in the X-ray range. Data from Reference 32.

X-ray reflection does not occur except for very small grazing angles, below the critical ≈(2*δ* )^1/2^ value, where 1 − *δ* is the real part of the index of refraction, 1 − *δ* − *iβ*. As seen in Figure 1 for the case of Au [10], the value of *δ* for “soft” X-rays (<1 keV photon energy) is in the 0.001–0.1 range, giving critical angles of 0.04–0.2 rad. For hard X-rays, *δ* is in the 0.0001–0.001 range, so that reflection only occurs for grazing angles smaller than a few hundredths of a radiant. The need to work at such small grazing angles greatly complicates the technical problems of optical devices based on reflection.

The small deviations from unity—*i.e.*, the small *δ*-values—of the index of refraction also complicate the technology of refractive optical components. Although both reflection and refraction devices are used for X-rays, the above difficulties orient the technology towards lenses based on interference, such as the Fresnel zone plates (FZPs) [33].

**Figure 2:**
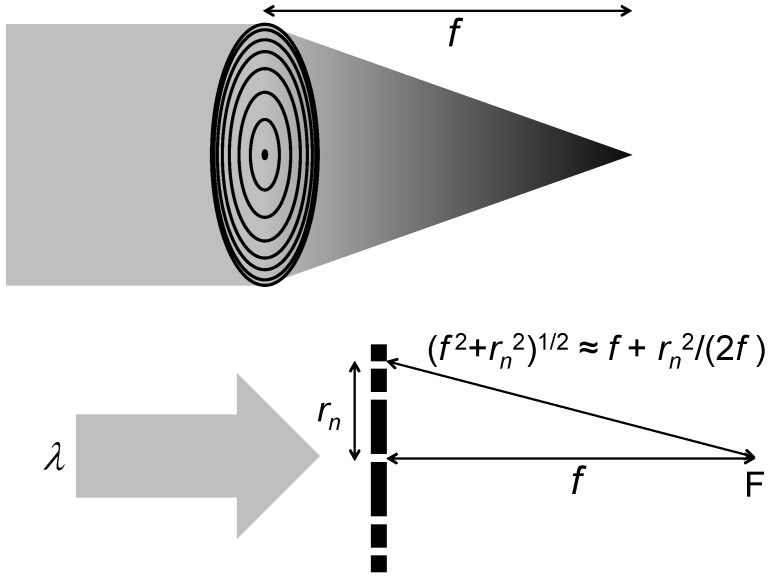
Scheme of a Fresnel zone plate (FZP).

The FZPs for X-rays are not conceptually different from those used for visible light. As seen in Figure 2, a standard (circular) FZP consist of a series of concentric circular zones, with alternating absorbing and transmitting zones. The focusing effect at the focal distance *f* is created by the constructing interference of waves passing through the transmitting zones.

As shown in the bottom part of Figure 2, the optical path of the wave passing through the *n*-th zone of radius *r_n_* and traveling to the focal point F is given by (*f* ^2^ + *r_n_*^2^)^1/2^. Constructive interference occurs if the optical path difference for adjacent zones equals the wavelength *λ*. Assuming *r_0_* = 0, this gives:

(1) $${\left(f^{2}+r_{n}^{2}\right)}^{1/2}-f=n\lambda$$

For small X-ray wavelengths, this condition requires *r_n_* << *f*, so that (*f* ^2^ + *r_n_*^2^)^1/2^ is approximately *f* + *r_n_*^2^/(2*f*) and Equation (1) becomes:

(2) $$r_{n}={\left(2n\lambda f\right)}^{1/2}.$$

This is the standard FZP focusing condition. Note that Equation (2) was derived with a second-order Mac Laurin expansion for *r_n_*/*f*. We neglected the next term of the expansion, which is quadratic and corresponds to the spherical aberrations [33].

Equation (2) has several important implications, the most relevant being the spatial resolution. According to the classic Rayleigh criterion, the theoretical spatial resolution of a lens is:

(3) Δx=1.220Nλ

where *N* = *f*/*D* is the f-number of the lens, *D* being the lens diameter. For a FZP, *D* = 2*r_m_*, where *m* is the maximum value of *n* and *r_m_* the position of the outermost zone. Therefore, *N* = *f*/(2*r_m_*) and, from Equation (3):

(4) $$\Delta x=1.220f\lambda /2r_{m}$$

Note that Equation (2) implies that *r_m_* is related to the (transverse) width of the outermost zone, Δ*r_m_*. In fact, assuming equal width for adjacent transmitting and absorbing zones, Δ*r_m_* equals the difference between the values given by Equation (2) for *n* = *m* and *n* = *m* − 1, *i.e.*,

(5) $$\Delta r_{m}={\left(2m\lambda f\right)}^{1/2}-{\left[2\left(m-1\right)\lambda f\right]}^{1/2}\approx \left\{\frac{\partial \left[{\left(2m\lambda f\right)}^{1/2}\right]}{\partial m}\right\}\times 1$$

and therefore, from Equation (4):

(6) $$\Delta x=1.220\Delta r_{m}$$

This result has fundamental implications on the FZP technology. The theoretical resolution is determined by the capability to manufacture narrow outermost zones. As we shall see, in recent years the benchmark values of Δ*r_m_* rapidly moved from the 100 nm level towards 10 nm.

Note that Equation (6) is valid for first-order focusing, *i.e.*, focusing at the distance *f* given by Equation (2). We shall see, however, that focusing also occurs for odd fractions of this value, *f*/3, *f/*5, *f*/7, *etc.*, although with decreasing efficiency. The corresponding f-numbers decrease as the focusing order increase, and so does Δ*x* with respect to Equation (6).

Equation (5) reveals another important consequence of the focusing condition, Equation (2). The diameter *D* = 2*r_m_* of the FZP is related to the outermost zone width:

(7) $$D=f\lambda /\Delta r_{m}$$

this means that the nanofabrication technology for FZPs affects not only the outermost zone width but also the diameter of the lens. The wavelength in the numerator of Equation (7) implies that FZPs for X-rays are much smaller than for visible light. The rather trivial technology for visible FZPs thus becomes a major nanofabrication challenge for X-rays.

We must now turn our attention to the FZP efficiency. This is the fraction of the incoming power that is focused by the lens. Even in an ideal situation, this fraction is small for reasons that can be qualitatively understood. First, the opaque zones of the FZP absorb part of the power. Second, some power is not focused at all or, rather, focused at an infinite distance. In fact, for an infinite distance the optical path difference between transmitting zones is always zero and therefore the interference constructive.

Third, focusing occurs not only for the distance *f* but also for the distances *f*/3, *f*/5, *f*/7 *etc.* In fact, the optical-path conditions valid for *f* (Equations (1) and (2)) also apply to integer fractions of *f*, but theory shows that only for odd integers there is power at the fractional focal points. Thus, the focused power is divided among all (odd) focusing orders. The reason can be also understood by considering the inverse optics case: the hologram of a point, corresponding to a single focusing distance (first order). The hologram would resemble the pattern of a FZP, but with smooth, sinusoidal-like oscillations between transmission and absorption. In a standard FZP, the transitions between zones are instead abrupt, and this produces higher-order focusing [33].

For such a standard FZP, two results are valid: (1) the maximum efficiency occurs when the widths of a transparent zone and that of the adjacent absorbing zone are equal; (2) the power fraction focused at the *q*-th order focal point is:

(8) $$E=1/{\left(q\pi \right)}^{2}\;\text{for}\;q=1,3,5\ldots \;\text{and}\;E=0.25\;\;\text{for}\;q=0$$

Thus, only 1/π^2^ ≈ 10% of the power is focused on the 1st order focal point.

This situation can be improved by several measures. In a “phase plate”, the absorbing zones are replaced by phase-shifting zones that change the phase of the waves by π. The corresponding theoretical first-order focusing efficiency becomes larger than 40%, essentially because there is no more zero-order focusing at an infinite distance but only power partition among odd-order focal points.

All the above results, however, are based on the assumption of perfectly transmitting and perfectly absorbing (or phase-reversing) zones, not quite realistic in the case of FZPs for X-rays. In his pioneering article of 1974, Kirz [34] injected realism into the analysis by considering actual fabrication materials with their specific *δ* and *β* values in the X-ray range. He thus found that with a suitable choice of materials and a suitable design (in particular, the zone thickness) the ideal efficiency can be increased up to 40%, essentially by decreasing the power lost to absorption and to the zero-order beam.

The properties of real materials explain in particular the fundamental technical differences between FZPs for soft X-rays (strong absorption) and hard-X-rays (very weak absorption). Consider for example a FZP whose absorbing zones have thickness *T* and are built with a material with a given *β*-value. The wave amplitude is not completely eliminated by the absorbing zones but attenuated by a factor exp(−4π*Tβ*/*λ*). Neglecting for the moment the phase modifications, this would changes Equation (8) to:

(9) $$E=\frac{1}{{\left(q\pi \right)}^{2}}\left[1+exp\left(-\frac{4\pi T\beta }{\lambda }\right)-2exp\left(-\frac{2\pi T\beta }{\lambda }\right)\right]\;\text{for}\;q=1,3,5\ldots$$

and

(10) $$E=0.25\left[1+exp\left(-\frac{4\pi T\beta }{\lambda }\right)-2exp\left(-\frac{2\pi T\beta }{\lambda }\right)\right]\;\text{for}\;q=0$$

In the limit case of infinite thickness and complete absorption, Equations (9) converge to Equation (8). But in general the limited absorption does play a role.

**Figure 3:**
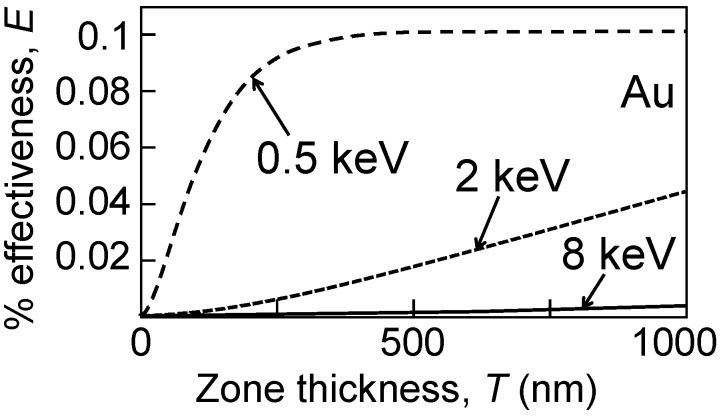
Theoretical Au FZP first-order effectiveness curves as a function of the thickness, given by Equation (9). Note that this approach is not realistic since it does not include phase effects.

Consider for example the case of a strong X-ray absorber like gold and three representative photon energies on the way from soft-X-rays to hard-X-rays: 0.5, 2 and 8 keV. The corresponding *β*-values are 4.76 × 10^−3^, 1.06 × 10^−4^ and 4.96 × 10^−6^ [10]. Figure 3 shows the corresponding plots of Equation (9) for first-order focusing, as a function of the absorbing zone thickness.

The effects of the weaker absorption at larger photon energies are quite evident: whereas at 500 eV the saturation efficiency is reached with a zone thickness of a few hundred nanometers, the same thickness produces only a fraction of that efficiency at 2 keV and 8 keV.

Equations (9) and (10) and Figure 3, however, are not realistic since they do not take into account the effects of the absorbing zones on the phase of the waves, described by the *δ*-value. Including such effects, Equations (9) and (10) change to:

(11) $$E=\frac{1}{{\left(q\pi \right)}^{2}}\left[1+exp\left(-\frac{4\pi T\beta }{\lambda }\right)-2exp\left(-\frac{2\pi T\beta }{\lambda }\right)\cos\left(\frac{2\pi T\delta }{\lambda }\right)\right]$$

for q=1,3,5… and

(12) $$E=0.25\left[1+exp\left(-\frac{4\pi T\beta }{\lambda }\right)-2exp\left(-\frac{2\pi T\beta }{\lambda }\right)\text{cos}\left(\frac{2\pi T\delta }{\lambda }\right)\right]$$

For q=0.

Note that the last term within square brackets can either decrease or increase the odd-order power depending on the phase shift, that determines the sign of the cosine factor. Taking the maximum values for the exponentials and the cosine, the first-order efficiency can surpass 40%: this is the already mentioned zero-order theoretical value for a pure phase plate.

**Figure 4:**
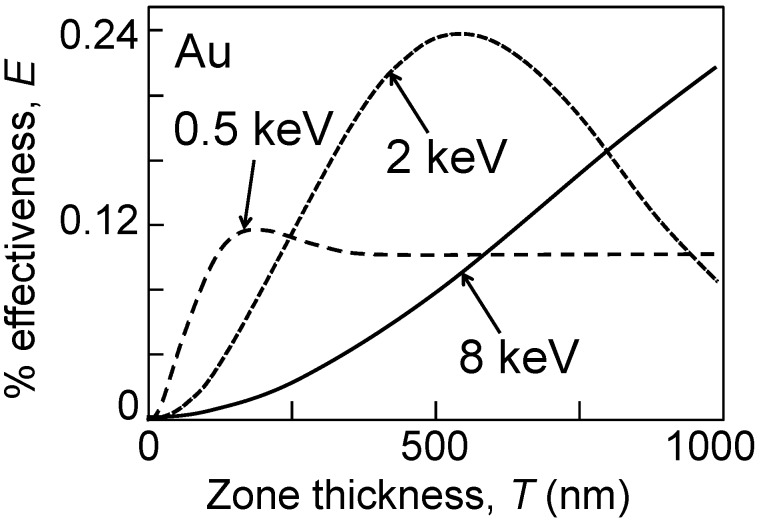
Theoretical Au FZP first-order effectiveness curves as a function of the thickness, given by Equation (11) which takes into account phase effects.

Figure 4 shows, again for Au, the plot of the first-order efficiency given by Equations (11) as a function of *T*. The Au *δ*-values for 0.5, 2 and 8 keV photons are 4.86 × 10^−3^, 5.10 × 10^−4^ and 4.77 × 10^−5^ [32]. The comparison with Figure 3 shows the strong effects of the phase changes, and in particular the possibility to reach efficiency values much larger than the previous asymptotic limit ≈10%. Once again, however, the limited absorption at high photon energies limits the efficiency for small thickness values.

The main implication of the above analysis is the increased technical difficulties in fabricating FZPs when the photon energy increases from soft to hard-X-rays. Higher efficiencies require larger thicknesses. However, high spatial resolution implies, according to Equation (6), a small outermost zone width. Thus, the combination of high resolution and high efficiency requires a narrow width together with a large thickness, *i.e.*, a large “aspect ratio” *T*/Δ*r_m_*.

The related technical problems are quite relevant. First, fabricating nanostructures with a high aspect ratio is *per se* a formidable challenge. Furthermore, the high aspect ratio implies mechanical stability problems that can jeopardize the practical use of the FZPs. Solving the nanofabrication and stability problems is the key issue in the technology of FZPs for hard-X-rays.

## 2. Fabrication Procedures: Examples

Over the past several years, a number of laboratories introduced innovative fabrication procedures for FZPs to tackle the aforementioned problems [33]. The fabrication strategies include electroplating the metal zones in a previously shaped polymer mold, dry etching with a hard mask and techniques based on atomic layer deposition (ALD) [11,12]. The first strategy, when applied to soft-X-ray FZPs made out of Ni, reaches 13 nm outermost zone widths [8]. In 2010–2011, dry etching could obtain 50 nm outermost zones [9] and atomic layer deposition plus sectioning (see below) could reach 35 nm [11]. This situation, however, evolves continuously. Furthermore, many variations of the standard approaches are being implemented [11,15].

**Figure 5:**
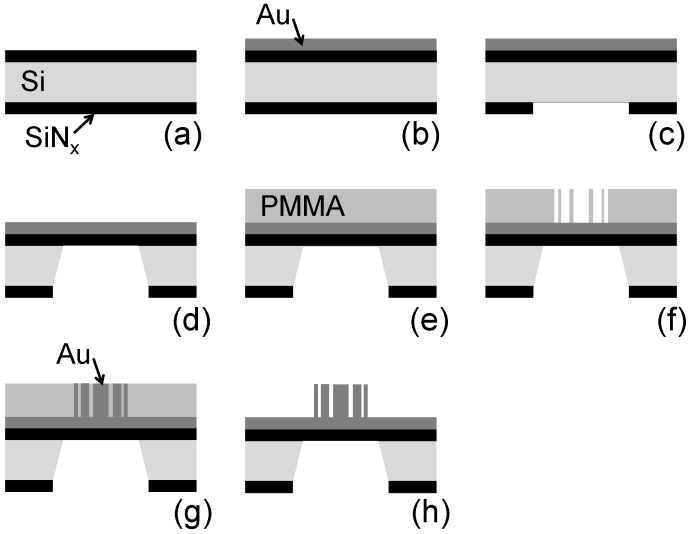
Schematic description of the FZP fabrication process based on e-beam writing and electrodeposition—see Reference 2.

A full review of this technical progress is beyond our scope. We would like to briefly present here two examples that give a good idea of the complexity of X-ray FZP fabrication. Figure 5 schematically shows the subsequent steps of a process involving electron-beam (e-beam) lithography and electrodeposition, yielding both a narrow outermost zone width and a high aspect ratio [2].

The process begins (Figure 5(a)) by coating both sides of a high-quality Si substrate with a 1 µm SiN*_x_* layer; the coating technique is low-pressure chemical vapor deposition (LPCVD). Then, thermal evaporation produced on one side only a 100 nm thick Au layer (Figure 5(b)).

The coated Si slab is cut into small (typically 7 × 7 mm^2^) squares and reaction ion etching (RIE) removes enough SiN*_x_* from the side without Au coating to open up a 3 × 3 mm^2^ window at the center of each square (Figure 5(c)). KOH is then used to remove the Si exposed by the window (Figure 5(d)); this leaves only the Au-covered SiN*_x_* layer.

Spin-coating is subsequently used (Figure 5(e)) to cover the Au overlayer with poly(methyl methacrylate) or PMMA, a photoresist material. The PMMA coating is from several hundred nanometer to more than 1 micron thick. A FZP pattern is written directly on the photoresist by e-beam lithography (with a typical operating voltage of 100 keV).

After PMMA development with a mixture of isopropanol and water and removal of the residues by ultrasounds, this produces the desired negative pattern (Figure 5(f)). Au electrodeposition inside a special cell then fills the trenches in the PMMA (Figure 5(g)); afterwards, the PMMA is chemically removed, leaving the high-aspect-ratio Au FZP, as shown in Figure 5(h).

The process by itself is not conceptually overcomplicated. However, reaching the performance for narrow outermost zones with a high aspect ratio and mechanical stability is difficult. In essence, each part of the process must be progressively optimized, mostly by a series of empirical steps [2].

Some relevant targets of this optimization are: the design of the pattern itself to improve mechanical stability; the identification of the optimal e-beam voltage and dose; finding the best type of PMMA and a suitable (and non-conventional) chemical agent to develop it; checking the PMMA pattern by electron microscopy of microsectioned specimens; identifying the most effective electrolyte; designing and building an *ad hoc* electroplating cell with features like degassing, stirring and filtration; identifying the ideal temperature and current density for the electrodeposition.

**Figure 6:**
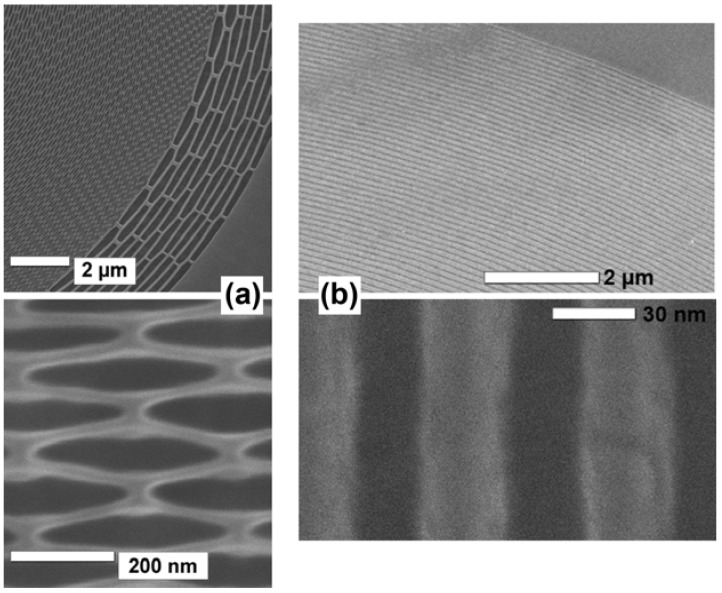
Examples of products of the fabrication process of Figure 5 [2]. (**a**) Scanning electron microscopy (SEM) images of a special “broken-line” FZP pattern at the stage corresponding to Figure 5(f); (**b**) SEM images of an Au FZP with 40 nm outermost zone width and an aspect ratio of 12.

The optimization of a subset of features does not work since the non-optimal ones dominate the performances. The parallel optimization does produce instead good results. We see in Figure 6 some examples [2]. Figure 6(a) is scanning electron microscopy (SEM) images of a specially designed FZP pattern at the stage of Figure 5(f). This “broken-line” design (with a characteristic sacrificial layer) markedly improves the mechanical stability compensating the problems caused by the high aspect ratio. Figure 6(b) shows an example [2] of final product: SEM images of an Au FZP with 40 nm outermost zone width and an aspect ratio of 12.

**Figure 7:**
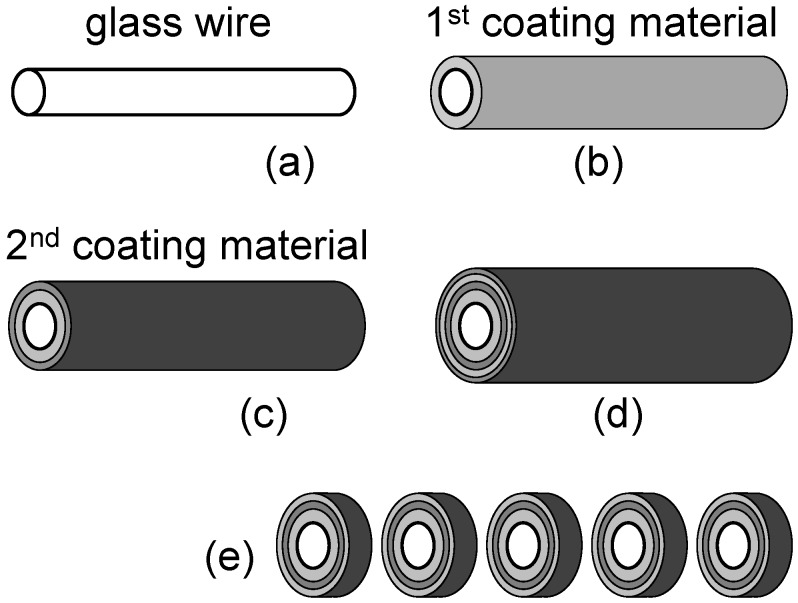
Schematic description of the FZP fabrication process based on atomic layer deposition (ALD) and ion beam sectioning—see References 10–12.

Figure 7 schematically explains the second example of fabrication strategy, based on ALD and ion beam sectioning [11,12]. ALD is a vapor phase deposition technique that exploits self-limiting surface reactions to achieve extremely high precision in the coating thickness. As seen in Figure 7(a)–(d), the first component is a thin glass wire (fiber) (a). Then coating begins with a first coating material (b), followed by a second (c), and continues sequentially (d) to produce the transversal FZP pattern. Finally, the coated wire is “sliced” (e) by ion beam sectioning producing precise and thick FZP with the desired aspect ratio.

Different variations of this multicoating approach were applied to the fabrication of FZPs both for soft-X-rays and hard-X-rays [11,12]. In the later case, very high aspect ratios can be achieved with reduced mechanical stability problems with respect to FZPs with vacuum between the absorbing zones. For example, Koyama *et al.* [12] reported in 2012 the fabrication of MoSi_2_-Si multilayer-coating FZPs with an outermost zone width of 40.4 nm and very high aspect ratio, optimized for 20 keV photons.

Note that with two different materials for adjacent zones Equation (10) must be modified taking into account the *δ* and *β* values of both materials:

(13) $$E=\frac{1}{{\left(q\pi \right)}^{2}}\left\{1+exp\left(-\frac{4\pi T\beta_{1}}{\lambda }\right)+exp\left(-\frac{4\pi T\beta_{2}}{\lambda }\right)-2exp\left[-\frac{2\pi T\left(\beta_{1}+\beta_{2}\right)}{\lambda }\right]\cos\left[\frac{2\pi T\left(\delta_{2}-\delta_{1}\right)}{\lambda }\right]\right\}$$

for =1,3,5… , and

(14) $$E=0.25\left\{1+exp\left(-\frac{4\pi T\beta_{1}}{\lambda }\right)+exp\left(-\frac{4\pi T\beta_{2}}{\lambda }\right)-2exp\left[-\frac{2\pi T\left(\beta_{1}+\beta_{2}\right)}{\lambda }\right]\cos\left[\frac{2\pi T\left(\delta_{2}-\delta_{1}\right)}{\lambda }\right]\right\}$$

for q=0.

This also enhances the flexibility in optimizing the FZP performances and in particular the efficiency [17].

The two methods discussed above (as well as others) are largely complementary and affected both by common and specific difficulties. The first delivered record spatial resolution but requires a complex parallel optimization. The second is more suitable for alternating materials and possibly for larger-scale production but does not yet reach extreme resolution levels. We thus expect both of them to play a significant role in the forthcoming years

## 3. Evaluating the Spatial Resolution

Lateral resolution is a benchmark parameter to assess the hard-X-ray FZP progress. Therefore, it is important to conceptually reconcile the different criteria used to define it. We briefly review here some of such criteria and their links.

We already mentioned (Equation (6)) that the ideal Rayleigh resolution is directly related to the outermost zone width Δ*r_m_*. One could thus use Δ*r_m_* as a measure of spatial resolution without further efforts. As we shall see, this can lead to overestimates.

Before discussing this point, we would like to remind the reader what is the Rayleigh criterion. Consider a circular lens of diameter *D* and focal distance *f*, used to image a point source of waves. The image is affected by diffraction: the radial intensity pattern is given by an Airy function, the positions of whose maxima and minima depend on *D* and *f*.

Consider now not one but two point sources at a lateral distance Δ*x*. The diffraction Airy patterns could prevent distinguishing the two points in the image if Δ*x* is too small. The Rayleigh criterion adopts as the lower limit the Δ*x* for which the maximum of one Airy pattern corresponds to the first minimum of the other. This leads to Equation (3) and therefore, for FZPs, to Equation (6).

However, the Rayleigh limit may not be a realistic assessment of the practical resolution if the signal level is too small and the noise too high: features resolved according to the Rayleigh criterion are not distinguishable in practice. Specifically, one must compare the noise to the contrast level. The actual resolution is thus a combination of optical parameters, signal level and contrast.

This suggests using practical evaluations rather than just the Rayleigh criterion. The simplest approach is to evaluate from the images the smallest visible features. This, however, can lead to overestimates, since features closer than the Rayleigh limit can still be distinguished from each other if the contrast is high and the noise is limited: the mere visibility of small features is a subjective criterion.

Another popular approach is using line scans, *i.e.*, intensity plots across the sharp edge of an image feature. Assume for simplicity an infinitely sharp edge and a Gaussian instrumental broadening—that transforms a point—object into an image whose radial intensity is proportional to exp[−*r*^2^/(2*σ* ^2^)]. The line scan is described by the convolution of this function with the step. The Gaussian broadening parameter *σ* can be derived by using the “10%–90%” criterion—*i.e.*, by measuring the distance between the points in which the intensity is 10% and 90% of the difference between maximum intensity and the background. This distance is indeed proportional to *σ*.

If the broadening is described by an Airy function, it can be approximated by a Gaussian. The relation between the two curves leads to the following expression for the Rayleigh resolution: Δx≈2.77σ; in turn, one can use this result to shows that the 10%–90% criterion approximately corresponds to the Rayleigh resolution Δ*x*.

**Figure 8:**
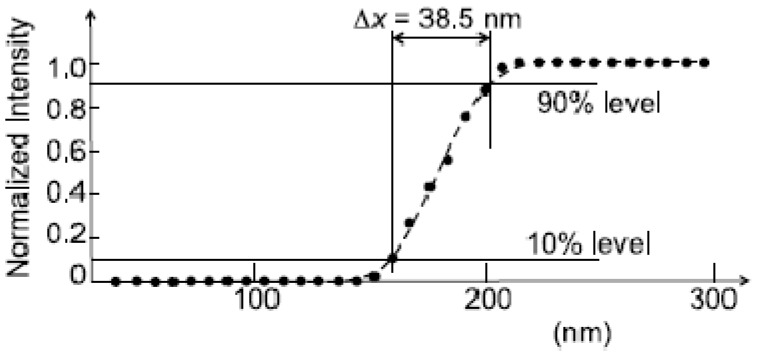
Spatial resolution evaluation by the line scan method using the 10%–90% approach that corresponds to the Rayleigh criterion. Data from Reference 4.

Figure 8 shows an example of this approach [4]. The conceptual problems are quite evident. First, the 10%–90% criterion assumes an infinitely sharp edge. If the object edge is not sharp, for example because of its morphology, its intrinsic broadening is convolved with the instrumental one. This leads to an underestimate of the Rayleigh resolution. Such a problem can be alleviated by scanning across many different edges in search for a minimum, which is closer to the Rayleigh resolution. The practical application of the 10%–90% criterion is also affected by the noise and by the corresponding uncertainty.

This brings up again the role of signal and noise. Two possible methods to include them in the resolution evaluations are the power spectrum analysis (PSA) [5,6,7] and the Modulation Transfer Function (MTF) approach [5], directly related to the “Rayleigh contrast criterion” [1].

The PSA of an image essentially consists [5,6,7] of calculating the two-dimensional Fourier transformation of the intensity in space, squaring it and integrating azimuthally over 2π. To avoid edge effects, the image intensity is preliminarily filtered with a window function such as the “Hamming” or “Hann” (raised cosine) windows. The analysis can be extended to a collection of images.

The Fourier-related spectrum contains components with high (spatial) frequency that correspond to small features. One can thus derive the high-frequency cutoff that corresponds to the smallest visible features. This limit can be caused either by the small size of the features or by the instrumental resolution. Therefore, it is a potentially pessimistic estimate of the latter.

Even if the cutoff is determined by the instrumental resolution, the relation of the value so obtained with the Rayleigh resolution is not straightforward. As already mentioned, features smaller than the Rayleigh resolution can still be visible if the noise is limited, and this is reflected in the Fourier analysis. The PSA values for resolution could therefore be artificially better than those based on the Rayleigh criterion. Still, they do provide a good idea of the smallest detectable features.

**Figure 9:**
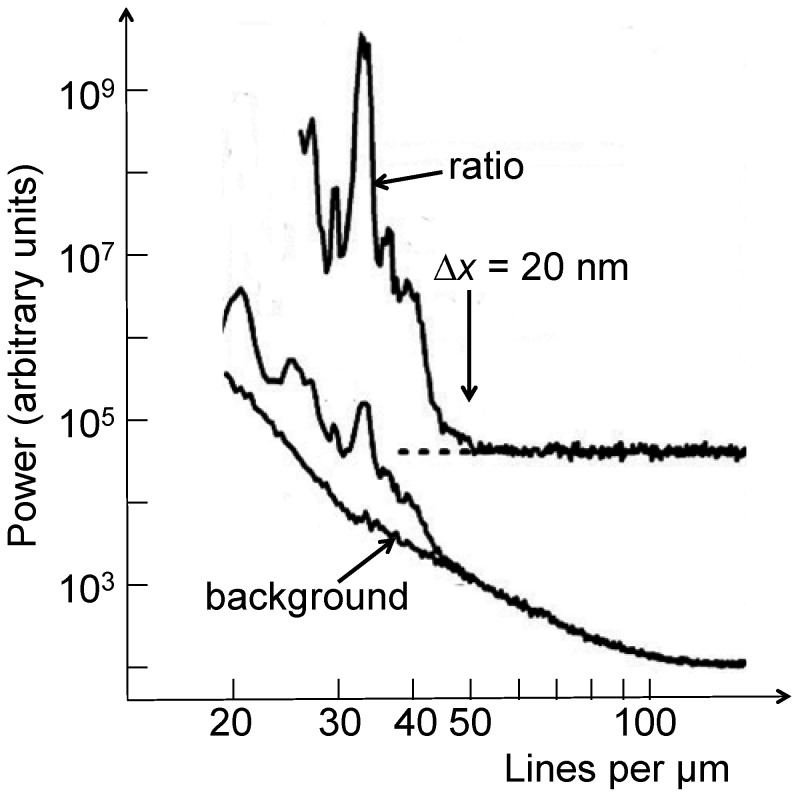
Spatial resolution evaluation by power spectrum analysis (PSA) [5,6,7]. Data from Reference 5.

Figure 9 shows an example of PSA, applied to an Au FZP with 30 nm outermost zone width [5]. We see the results from two image regions, one with features and the other a featureless background. The ratio of the two curves facilitates the identification of the cutoff, indicating a minimum detectable feature size of 20 nm—whereas the estimated Rayleigh resolution was ≈ 29 nm.

**Figure 10:**
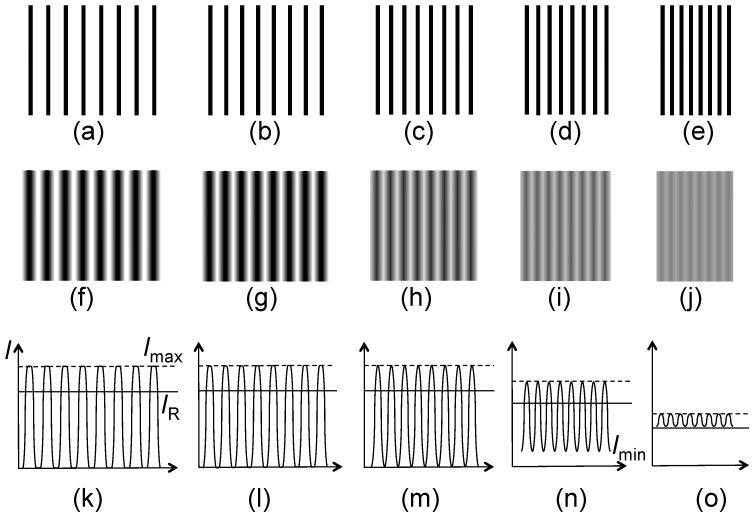
Schematic explanation of the modulation transfer function (MTF) method to derive the spatial resolution [5]. (**a**)–(**e**): Series of hypothetic objects consisting of ultrasharp lines with increasing spatial frequency; (**f**)–(**j**) The corresponding images with increasing instrumental blurring; (**k**)–(**o**) Intensity plots showing the minimum and maximum intensity values, *I_max_* and *I_min_*, used to define the MTF and the Michelson contrast *C*. The solid horizontal line in (**k**)–(**o**) (labeled as *I_R_* in (**k**)) is, for each figure, the value of *I_min_* corresponding to the Rayleigh criterion.

**Figure 11:**
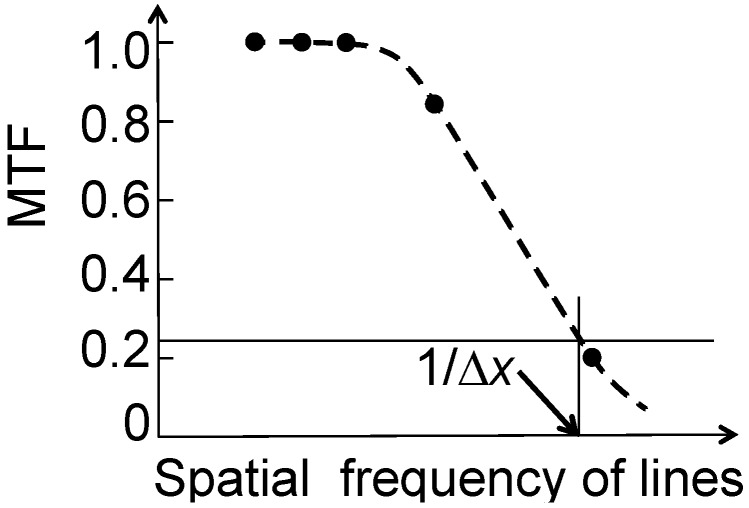
Practical example of the MTF method of spatial resolution evaluation. Simulated data from Figure 10.

Concerning the MTF and “Rayleigh contrast” approach, Figure 10 schematically illustrates a hypothetic case: the instrumental blurring of images of periodic, infinitely sharp series of lines. The blurring effects become more pronounced as the distance between adjacent lines decreases. The phenomenon can be quantified by defining the MTF as (*I_max_* − *I_min_*)/*I_max_*, where *I_max_* and *I_min_* are the maximum and minimum detected intensities. The MTF is then plotted as a function of the spatial line frequency, as shown in Figure 11.

The link with the Rayleigh criterion is established by noting that the MTF value given by two Airy patterns for a distance corresponding to Δ*x* is ≈0.265 (*i.e.*, *I_min_*/*I_max_* ≈ 0.735). As shown in Figure 11, this makes it possible to derive Δ*x*. The same approach can be implemented for images of non-periodic small features.

Another version of this approach is based on the notion of Michelson contrast [1], defined as *C* = (*I_max_* − *I_min_*)/(*I_max_* + *I_min_*). The Rayleigh-limit value of *C* is (1 − 0.735)/(1 + 0.735) ≈ 0.153—see again Figure 11. In both cases, the evaluation gives a resolution Δ*x* that is numerically linked to the Rayleigh criterion, but not equivalent to it in terms of the physical effects. Hence, one should use for this value the term “Rayleigh contrast resolution” rather than “Raylegh resolution”.

**Figure 12:**
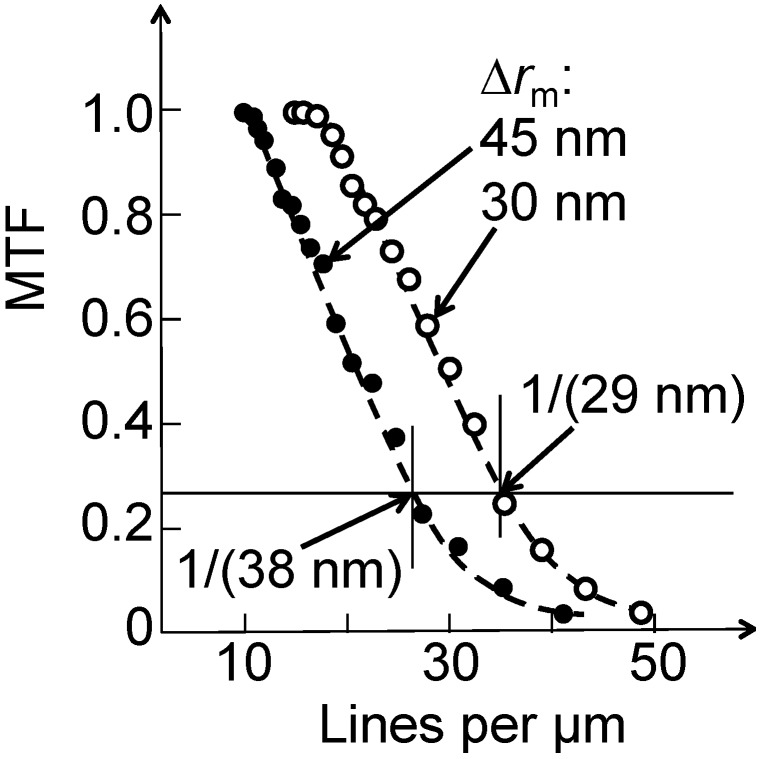
A real case of MTF analysis for two FZPs with different outermost zone widths, 30 nm and 45 nm. The marks show the extracted spatial frequencies, corresponding to 29 nm and 38 nm resolution. Data from Reference 5.

**Figure 13:**
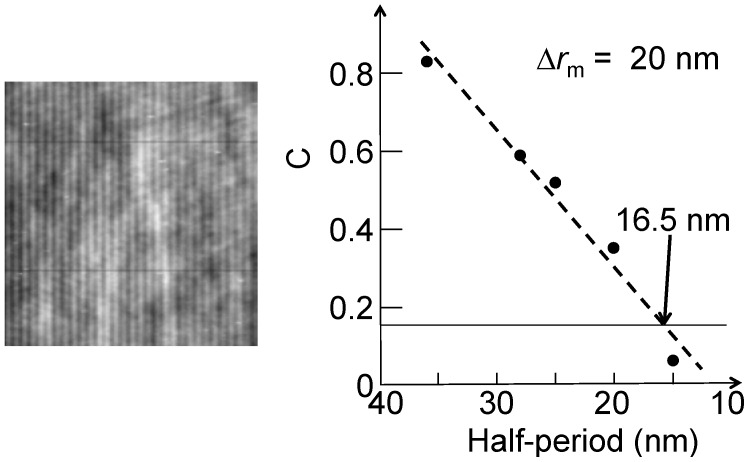
A real case of Rayleigh contrast resolution analysis. Data from Reference 1.

The advantage of using the MTF or *C* is the automatic evaluation of the noise effects when measuring the intensities *I_max_* and *I_min_*: Such effects are thus somewhat taken into account, contrary to the straight Rayleigh criterion. Figure 12 [5] and Figure 13 [1] show two real examples of use of MTF and C to derive the Rayleigh contrast resolution of two different Au FZPs at 8 keV photon energy.

Besides the above image-analysis approaches, important progress was recently made in the evaluation of FZP focusing characteristics that determine the spatial resolution, using coherent imaging techniques. The approach can be traced back to Quinley *et al.*[35] and the evolution has recently accelerated [36,37]. A highly coherent source is used to obtain ptychographic data from a suitable strongly scattering object [37]. Powerful reconstruction algorithms yield not only the image characteristics but also those of the FZP focusing geometry. This type of method thus overcomes the theoretical and practical limitations of traditional image-analysis approaches, whose applicability to narrow focusing already reaches the limit.

## 4. Towards New Spatial Resolution Records

The progress towards better resolution level was quite spectacular in the most recent years. Such a progress has important implications: the new resolution levels open up entirely new domains of research. In particular, they bring into the scope of hard-X-ray microscopy biomedical areas such as neurobiology [1] and tumor angiogenesis [13].

**Figure 14:**
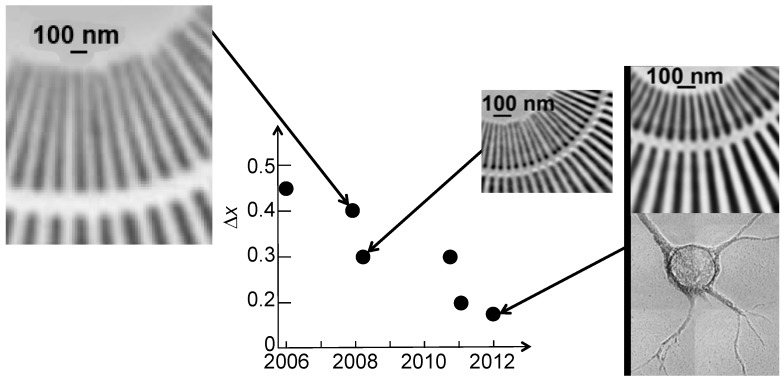
The progressive improvement in recent years of the spatial resolution for Au hard-X-ray FZPs. Data and pictures from References 14, 4–7 and 1 for the years 2006, 2008 (two sets), 2011 (two sets) and 2012.

Figure 14 provides a synthetic view of the recent progress for the specific case of high-aspect-ratio Au FZPs [1,4,5,6,7,14]. For the starting point, we refer to the report [14] by Xradia and its partner institutions in 2006 of a hard-X-ray Au FZP with outermost zone width of 50 nm, yielding a MTF resolution of 45 nm. The same tests demonstrated a resolution of 27 nm for the third-order focusing.

In the subsequent five years, the activities [1,2,3,4,5,6,7] of a collaboration centered at the Taiwan Academia Sinica and involving scientists from Switzerland, France, the USA and other countries improved the record resolution by a factor of three, while keeping the aspect ratio at high levels and guaranteeing the mechanical stability of the lenses. The products were rapidly transferred to real research and produced a number of interesting results [1,3,4,5,6,7,13].

What was the crucial factor in this progress? It is difficult to single out one element: the progress was produced by continuous optimization and cross-optimization of several different process components of the fabrication process, a “e-beam plus electrodeposition” approach. There is no indication that the final optimization was achieved, so there is still room for further improvements.

**Figure 15:**
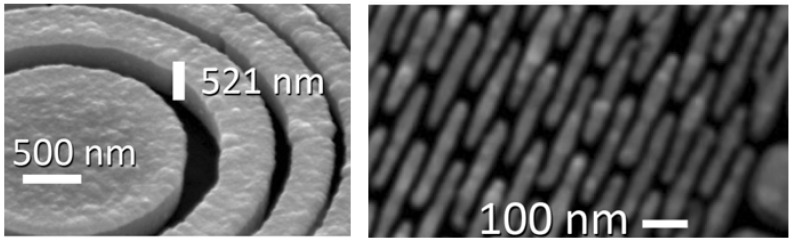
SEM images (from Reference 7) of a recent Au hard-X-ray FZP.

**Figure 16:**
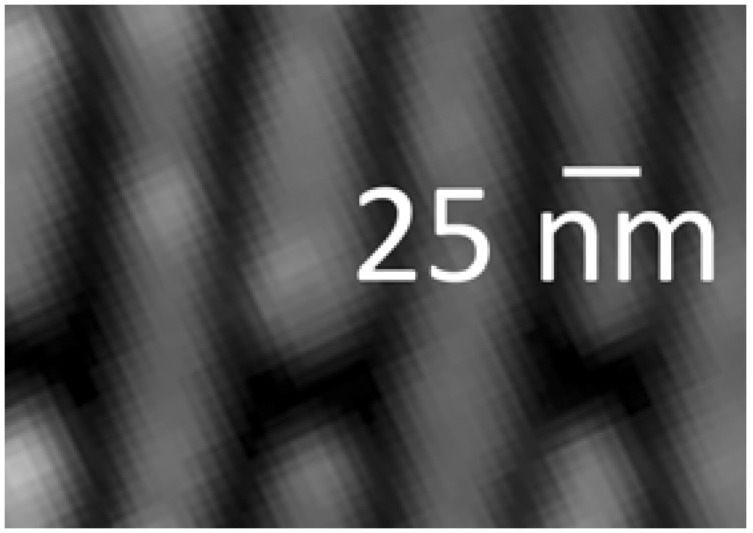
Zoomed picture corresponding to Figure 15 showing a width of 25 nm for the outermost zones [7].

Figure 15 and Figure 16 show examples [7] of the FZPs produced by this continuous progress. Note in particular the thickness of the Au structure in Figure 15 that corresponds to the high aspect ratio required for hard-X-rays.

At the forefront of FZP resolution, C. David and his co-workers recently used the “zone doubling” technique to fabricate FZPs capable to distinguish features separated by 12–15 nm [38,39]. This technique consists of first fabricating a silicon FZP template and then accurately coating it with a metal overlayer—such as iridium—with a much higher refractive index. Because of the different optical response of the two materials, this is equivalent to halving the effective zone period. So far, effective line widths down to 15 nm were announced, but the technique has the potential to go well beyond these levels.

## 5. Enhancing the Images: Contrast Agents and Phase Contrast

The fabrication technology is certainly a key ingredient in the hard-X-ray FZP progress, but not the only one. The capability of an image to deliver information is determined by several factors, also including contrast and noise. In the end, an excellent spatial resolution is not useful if not complemented by sufficient contrast to really detect the smallest features.

The use of contrast agents is a standard practice in imaging for the life sciences. However, contrast agents used for other microscopy are not easy to transfer to hard-X-ray microscopy—and require specific research and optimization efforts [40,42]. We would like to mention, in particular, the search for a suitable procedure in the case of neurobiology specimens [1].

The main problem in this case is the perfusion of the contrast agent (staining ingredient). In optical microscopy, fluorophores are commonly used and their penetration depth is adequate for the corresponding thin specimens, typically tens of microns thick. But this penetration depth is not sufficient for hard-X-rays, whose specimens must be considerably thicker. Furthermore, the fluorescent ingredients lack the heavy elements required to increase the hard-X-ray absorption for contrast enhancement.

A broad search [1] finally yielded a suitable staining procedure for hard-X-rays. The contrast agent is the same as in the Golgi-Cox method and is based on mercury and silver. However, the required incubation time to reach high penetration is much longer than for the standard Golgi-Cox procedure, and the temperature higher. Indeed, good penetration is achieved by increasing the temperature to 50 °C and by extending the incubation time to one month or even more. The augmented perfusion produces staining over the entire depth of the specimens, suitable for hard-X-ray imaging [1].

A complementary way to enhance contrast is to use phase effects in addition to absorption differences. A standard method to achieve phase contrast is the use of a Zernike phase ring [6]. Could this technique, however, be successfully used for hard-X-ray microscopy?

**Figure 17:**
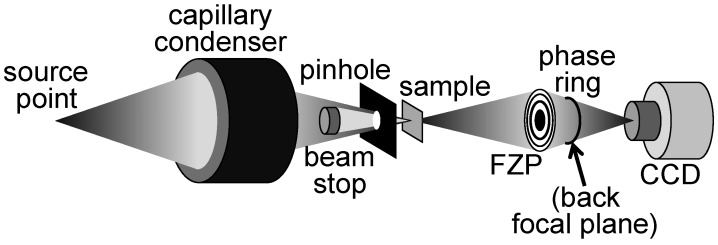
Schematic diagram of a hard-X-ray FZP microscope with the option of Zernike phase contrast—see References 6 and 7.

**Figure 18:**
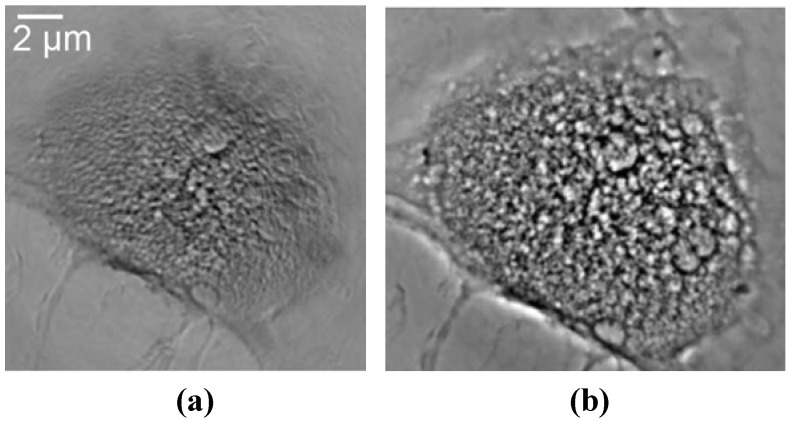
Comparison of an absorption-contrast micrograph (**a**) and of a Zernike phase-contrast micrograph (**b**). Data from Reference 6.

**Figure 19:**
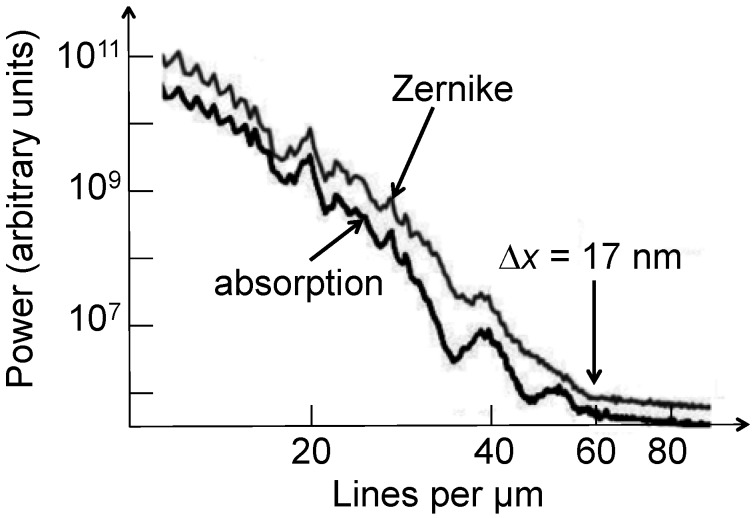
PSA method applied to Zernike-contrast and absorption-contrast images, indicating comparable levels of spatial resolution. Data from Reference 7.

Results like those of References 6 and 7 provided a positive answer. Figure 17 illustrates how a Zernike phase ring can be included in a hard-X-ray microscope based on a FZP [7]. Figure 18 shows the striking improvement produced by the ring as far as contrast is concerned [6]. Tests like those of Figure 19 [7] provide evidence that the improved contrast is not reached at the expense of resolution. Thus, Zernike phase contrast can be routinely used in hard-X-ray microscopy as well as in other imaging techniques.

## 6. Overview of Recent Progress

As in many other rapidly moving domains, one can distinguish here between three different types of progress: evolutionary, innovative and potentially groundbreaking. In the first class, we can note the steady improvement of the efficiency for one-component FZPs. For example, 5%–6% efficiency at 8 keV photon energy was reported in 2011 by Gorelick *et al.* [15] for Au FZPs with an outermost zone width of 50 nm and 500 nm thickness (aspect ratio 10) and by Chubarova *et al.* [10] with an outermost zone width of 50 nm and 570 nm thickness (aspect ratio 11.4).

The technique of stacking FZPs on top of each other was initiated sometimes ago (see for example Feng *at al.* [16]). The objective, of course, is to increase the thickness and the aspect ratio circumventing the mechanical stability problems. In 2010, Werner *et al.* [17] presented important progress in the use of this technique for nickel FZPs. In 2011, Kagoshima *et al.* [18] presented interesting results with “tandem” tantalum FZPs reaching a combined thickness of 4.8 µm.

Quite innovative is the technique of combining FZPs and two-dimensional gratings for phase contrast imaging: Berujon *et al.* [19] presented interesting results in 2012. The combination of the two elements notably allows the fast recovery of sample-induced phase shifts with less artifacts than, for example, a one-dimensional interferometer. Equally innovative was the approach to deal with thermal instability, successfully adopted by Nilsson *et al.* [20]. In 2011, these authors reported on the fabrication of very stable tungsten FZPs with excellent properties. 

Moving now to groundbreaking events, we would like to mention two groups of results. First, the implementation of hard-X-ray microscopy with a low-brilliance laboratory source, reported in 2011 by Kuwabara *et al.* [21]. Second and in the opposite direction, the first steps towards hard-X-ray microscopy with free electron lasers [31].

Free electron lasers deliver peak brightness levels higher than other sources by several orders of magnitude, and full spatial coherence in the entire spectral range relevant to our discussion. The advantages for imaging in general and X-ray microscopy are obvious and multi-faceted.

However, the price to pay is high: free electron lasers require FZPs of exceptional stability, capable of withstanding the very high peak intensity of the short FEL pulses. Quite significant in that regard is the progress in diamond-based FZPs, reported for example by Uhlén *et al.* in 2011 [22]. Also in 2011, David *et al.* [23] reported the first tests with diamond FZPs on an FEL, at the Linac Coherent Light Source (LCLS). Likewise based on diamond was the technique presented in 2010 by Wojcik *et al.* [24]: these authors used ultrananocrystalline diamond to prepare molds for the fabrication of Au FZPs by electroplating.

**Figure 20:**
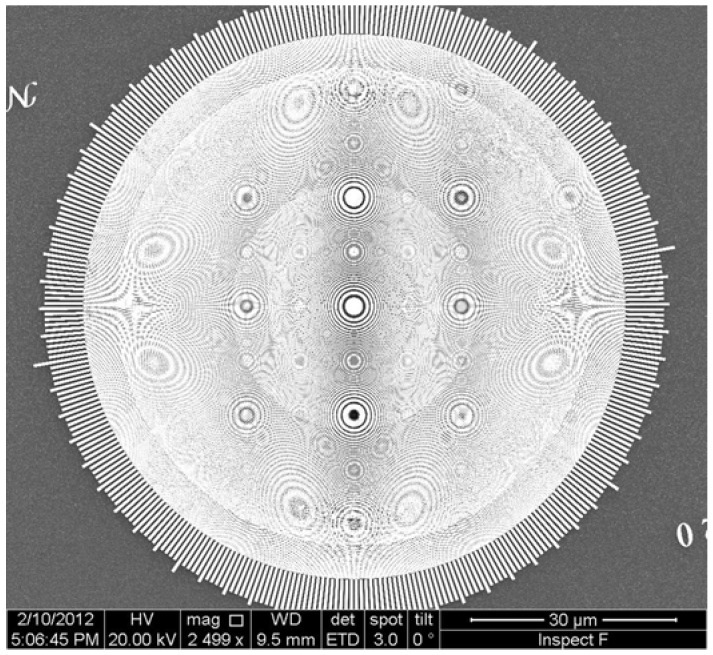
Overall pattern of one of the most advanced hard-X-ray FZPs of today, with an outermost zone width of 20 nm [1].

**Figure 21:**
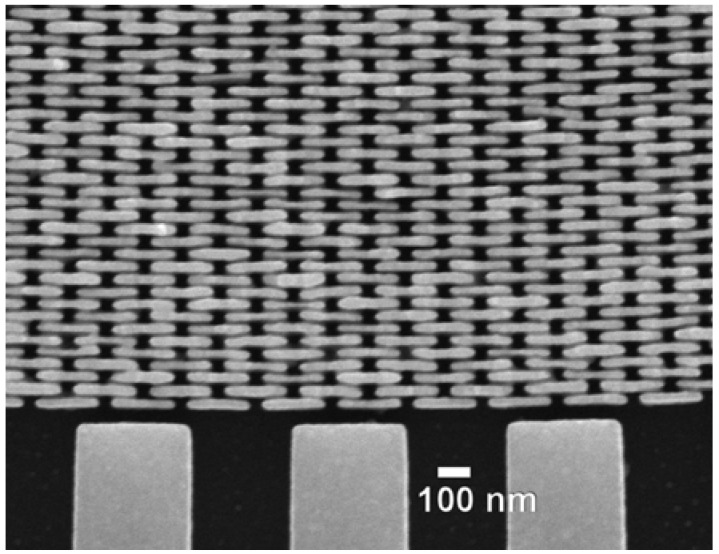
The region near the edge of the hard-X-ray FZPs of Figure 20. The “broken ring” pattern is designed to enhance the mechanical stability for high aspect ratios.

**Figure 22:**
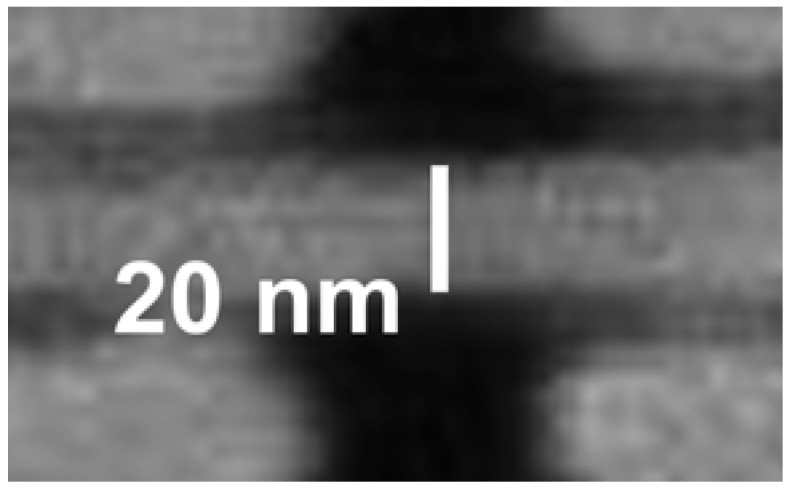
Magnified picture of a feature at the edge of the hard-X-ray FZPs of Figure 20 and Figure 21.

Many other developments could be mentioned [see for example References 25–29], but our scope here is not a comprehensive review. Rather, we would like to give the reader a dynamic idea of the ongoing developments. We thus believe that the best way to conclude our presentation is to show pictures of a most advanced X-ray FZP of today—see Figure 20, Figure 21 and Figure 22—knowing very well that the evolving technology will surpass them in the near future. Because of this progress, in the 12th decade after Roentgen’s discovery of X-rays radiology on the scale of cells and nano-objects is, finally, a wonderful reality!
